# When female circumcision comes to the West: Attitudes toward the practice among Somali Immigrants in Oslo

**DOI:** 10.1186/1471-2458-12-697

**Published:** 2012-08-27

**Authors:** Abdi A Gele, Elise B Johansen, Johanne Sundby

**Affiliations:** 1 Department of Social Science, Oslo University College, Pilestredet 35, Oslo, 0167, Norway; 2 Department of General Practice and Community Medicine, Section for International Health, University of Oslo, Oslo, Norway; 3 Section for Reproductive Health & Research, World Health Organization (WHO), Geneva, Switzerland

## Abstract

**Background:**

Female circumcision (FC) has lifelong adverse social and health consequences for women, and its abolition will not only enhance the health of children and women, but also promote gender equality. Like many other Western countries, Norway hosts a large proportion of immigrants from FC-practicing countries, though primarily from Somalia, which is the country with the highest prevalence of FC in the world. A behavioral change by the practicing communities has the best chance to successfully and sustainably eliminate this practice. However, FC prevention programs require a behavioral surveillance that monitors the process of change, with this being the first quantitative study since the major migration of the Somali community to Norway began in 1991 to investigate whether or not Somali immigrants’ attitudes toward the practice has improved in favor of its abandonment.

**Methods:**

A cross-sectional study using a respondent-driven sampling (RDS) was conducted in Oslo from April to June of 2011. A sample of 214 persons was interviewed, using structured questionnaires.

**Results:**

The results show that 70% of Somalis in Oslo support the discontinuation of all forms of FC compared to 30% who support its continuation, with the latter group more likely to be people who lived in Norway ≤ 4 years. Of the 10 girls who came to Norway at the age of ≤ 7 years, only one was circumcised, though whether the circumcision occurred before or after the girl’s arrival in Norway remains unclear. The perception that FC is required by religion was the sole factor to be significantly associated with an ongoing support of FC.

**Conclusion:**

The study reveals that Somalis in Oslo demonstrate a trend to abandon this practice over time. Nevertheless, the 30% of the people who still support its continuation, and who are primarily newly arrived immigrants, require a targeted intervention that is implemented in the early phase of the immigrants’ arrival.

## Background

On a global basis, an estimated 140 million women have been subjected to FC, with three million girls being at risk for the practice every year [1], the majority of whom live in 28 African countries and pockets in Asia [1]; however, FC has gone far beyond its traditional borders through migration, and as a result of political unrest in some FC-practicing countries in both Africa and Asia, many Western countries have confronted a large influx of immigrants from countries with FC traditions. Among these immigrants are people from Somalia, who currently form the largest ethnic group from FC-practicing countries in Scandinavia, the Netherlands and the UK [2-6]. Going from a country where being uncircumcised is an unspeakable shame to a new country where FC is illegal and stigmatizing raises a question of whether or not attitudes toward FC among Somali immigrants have changed in accordance with their new host’s culture.

Female genital mutilation (FGM), also known as female genital cutting (FGC) or female circumcision (FC), which is the term we use in this paper since it relates better to the local term used by the study’s participants, is a traditional surgery involving the removal or injury of important external female genitals for non-therapeutic reasons [7]. The World Health Organization (WHO), who use the term FGM, has classified it into four main types [7]. Type I involves the partial or total removal of the clitoris and/or the prepuce, while Type II involves the partial or total removal of the clitoris and labia minora. Being the most radical form, Type III involves the partial or total removal of the external genitalia and a sealing of the vaginal opening, leaving only a small hole for urine and menstrual blood to pass (whether with or without cutting the clitoris). Type IV involves all other harmful procedures to the female genitalia for non-medical reasons.

The victims of this practice suffer permanent and irreversible tissue damage that may negatively affect the health of the girls over the course of their entire lifetime [8]. The immediate problems that girls may experience during the procedure are extreme pain, excessive bleeding, shock, infections and death [1]. Other obvious complications also include the retention of urine, difficulties in menstruation and impaired sexual pleasure [9]. The WHO has documented the association between FC and an increased risk of birth complications, which may affect both the mother and child [10]. These complications include postpartum hemorrhage, extended maternal hospital stays, infant resuscitation, stillbirths, neonatal deaths and inpatient prenatal deaths [10]. Additionally, increased risks of cervical cancer [11] and death have also been associated with this practice [12].

Since the early 1990s, FC has gained an increased amount of international recognition as a violation of human rights, including the right to health. The United Nations Convention on the Rights of the Child, Article no. 24, obliges all States to abolish traditional practices that are harmful to the health of children [13]. In 1997, the World Health Organization (WHO) issued a joint statement in conjunction with the United Nations Children’s Fund (UNICEF) and the United Nations Population Fund (UNFPA), which described the public health and human rights’ implications of the practice, and recommended its elimination. As a result, the practice has been outlawed in many FC practicing countries as well as countries in Western Europe and the US. Moreover, the US policy also requires all African countries receiving US aid to outlaw the practice and begin awareness programs to eradicate FC entirely [14].

Somalia is part of the Horn of Africa, with a population of 7.4 million. FC has been universally practiced by the Somali people since time immemorial, though exactly where and how it came to Somalia is still unknown. Girls are often circumcised by a traditional practitioner, but the number of health professionals performing the practice is also increasing in Somalia [15,16], with approximately 90% of Somali girls experiencing the most radical form of circumcision, locally referred to as Pharaonic (Type III) [15]. The age of circumcision in Somalia is often before the age of 10 [17], with the average age ranging from 6.9 to 7.5 years [18,19]. The reasons for carrying out circumcision vary from doing it simply because others have done it, to a perception that FC is a religious requirement. Prior studies have reported on a universal prevalence of FC (100%) among Somali women [17,18,20], and according to Dirie and Lindmark, out of the 290 women who participated in their study, all of them had intentions to circumcise their daughters [20]. Furthermore, universal support for the continuation of the practice was reported from Somalia [17], where to circumcise a daughter or not is independent of one’s socio-economic status, with an equal prevalence seen among rich, poor, educated, uneducated, urban and rural peoples [15].

Following the onset of the civil war in 1991, approximately 25% of Somali people migrated from their home country, and are currently living either in neighboring countries as refugees or have resettled in Western countries that host immigrants. As a consequence of this migration, 30,000 Somali immigrants now make their home in Norway, thereby constituting the largest population of African immigrants [21], and thus the largest group from any FC-practicing country. Legislation prohibiting FC was introduced in Norway in 1995, with the law against FC stating that, “any person who willfully performs a procedure on a woman’s genital organs that injures or permanently changes the genital organs will be penalized for female genital mutilation. The penalty is a term of imprisonment of anywhere from 3 up to 6 years” [22]. The Norwegian action plan against FC was established in 2000, with the rationale of the action plan being to prevent FC and accomplish a behavioral change towards the practice [22]. The prevention policies consist of awareness raising, providing information to the members of the communities concerned and the public at large, as well as the education and training of medical professionals, health care workers, teachers and the empowerment of women.

However, the continued surveillance of attitudes toward the practice among immigrants from FC-practicing countries holds a paramount importance for achieving an understanding of the process of social change, in addition to the dynamics of FC. Even so, due to the lack of a sampling frame for immigrants, most of the studies on FC among immigrants in Western countries have been qualitative. To the best of our knowledge, there has only been a single quantitative study previously published in a peer-reviewed journal that has investigated attitudes toward FC among Somali immigrants [6]. However, the study had major limitations, as it first focused on attitudes of youth towards FC, and not adult populations. Second, it used a non-probability sampling, namely snowball sampling; hence, the sample was not representative and the results not generalizable. Therefore, this is the first quantitative study using a respondent-driven sampling (a probability sampling method) that investigates attitudes toward FC among immigrants in the West of different ages and gender.

### Theories of behavioral change

Theoretical models of behavioral change are needed to understand why and how interventions cause change. Accordingly, different theories were employed in relation to attitude and behavioral changes toward FC. Some researchers adopted stages of change theory [23], others used social convention theory [24], whereas Bettina Shell-Duncan used modified stages of change theory [25]. The stages of change theory were originally formulated on assessing behaviors based on individual decisions that did not take into account behaviors based on collective decisions such as the practice of FC. For this reason, Shell-Duncan modified the stages of change theory to help fit it to the practice of FC in which the decision regarding the abandonment of the practice is not in the hands of any one individual, but rather with a group of decision makers. According to modified stages of change theory, people abandon the practice when they obtain a motivation to end the practice and have the ability to act upon their decision, i.e. when the interconnected communities agree to stop FC because of a realization of its harmful effect. Moreover, Shell-Duncan argued that in some situations, people may abandon the practice even if they personally want it to continue, e.g. when other decision makers or social pressure force them to stop the practice or where there is an enforced law that forbids the practice with a subsequent fear of prosecution for breaking the law [25]. Social convention theory, which is the theory most used in FC research [24,26], postulates that FC can be terminated through the existence of a group of parents who choose not to circumcise their daughters [27]. A reduction in the prevalence of FC is therefore the ultimate indicator of a sustained change [28]. This study examines whether or not Somali immigrants’ exposure to Norwegian culture, with its different view towards FC, has altered immigrants’ attitudes and behaviors toward their longstanding tradition of FC.

## Methods

A cross-sectional study using respondent-driven sampling (RDS) was conducted in Oslo from April to June of 2011. The RDS consists of an enhanced snowball sampling, in which information on who recruited whom and the individual social network size providing a basis for the calculation of inclusion probability and minimally biased population indicators, as well as the variability of these indicators [29,30]. The RDS was initiated by small number of purposely recruited seeds, who were given uniquely coded coupons and incentives to recruit their peers to participate in the study. Eligible recruits who participated in the study and redeemed their coupons created the first wave of respondents. The first wave further recruited their peers, who then formed the second wave, with the cycle continuing until the predetermined sample size was achieved. The recruitment was motivated by primary incentives paid to participants for taking part in the survey, in addition to a secondary incentive for the recruitment of their peers [29]. RDS is largely used on behavioral studies [31], and is widely acknowledged for its potential in studying immigrant communities whose sampling frame is unavailable [32-35]. In the present study, we have chosen RDS as the best method for accessing Somalis in Oslo since they form a hidden group dispersed throughout a town with a population size of almost 600,000. While the sample frame of this immigrant group was unavailable to the researchers, Somalis in Oslo are closely networked, thereby indicating that RDS is an appropriate sampling method to use with them. RDS was further selected in order to accomplish a representative sample of this hard-to-reach community and to enable any needed adjustments for any recruitment bias [30].

### Recruitment process

Eligibility criteria for the study included being a Somali immigrant permanently and legally residing in Oslo, aged ≥18 and being willing and able to provide informed consent. Initially, a formative study involving 11 Somalis was conducted with the aim of understanding the social network structure of Somali immigrants, the average peer that each recruiter can recruit and the recruitment incentives necessary to motivate Somalis to participate in the study. A respondent-driven sampling design suggests that seeds should be persons who are socially connected and motivated to recruit others [29]. Thus, five eligible seeds, comprising socially well-connected individuals, were purposely selected based on a diversity of gender, location, years of stay in Norway and age. After providing informed consent, the seeds underwent an interview, were educated on how to refer other eligible Somalis, and were given two uniquely coded coupons to help refer their peers in their social network. The reason for only using two coupons was to elongate the recruitment waves so that the diversion of subsequent waves from the initial seeds was increased. Each seed proceeded to recruit two persons into their network, which became the first wave, with the first-wave participants further recruiting their peers, which then became the second wave. Each participant was compensated with 100 Norwegian kroner (NOK) for participating in the study, and received an additional 50 NOK for each recruited peer who met the eligibility criteria and participated in the study. The chain referral process continued until we obtained the desired sample size of 214, which was calculated using the formula necessary to determine the sample size required for estimating the sample proportions. The study was ethically approved by the Norwegian Regional Committee for Medical and Health Research Ethics (REK).

### Indicators

The questionnaires included both socio-demographic details and questions that assessed people’s knowledge and attitudes toward FC. The dependent variable was whether one supports the continuation or discontinuation of one form and/or all forms of FC. This question was categorized into four categories: I support the continuation of the Sunna form (Types 1 and 2); I support the continuation of the Pharaonic form (Type 3); I support continuation of both forms and I support the discontinuation of all forms of FC. During the analysis, the first three categories were collapsed together and coded as “1,” while those who supported the discontinuation of all forms of FC were coded as “0.” The knowledge questions included questions that addressed the health effects of FC, i.e. whether FC led to bleeding, infection, complications in child birth, difficulties in urination and the possible transmission of HIV, with the questions categorized as either “yes” and “no.” Questions that assess the attitude towards FC included: Is FC a religious requirement? Is it a harmful culture? Does it protect the honor of the girls? Does it prevent adultery? Does it lead to trustworthy marriage, etc.? All of these questions were categorized into four categories: yes for the Sunna form (Types 1 and 2); yes for the Pharaonic form (Type 3) and yes for both forms and none of the forms. Moreover, female participants were asked if they themselves had been circumcised, as well as the type of their circumcision, which was categorized as either S*unna* or *Pharaonic*. By contrast, male participants were asked if they would prefer to marry women who were circumcised or uncircumcised, while both male and females were asked if they intended to circumcise their daughters and the reasons behind their decision. An analysis of RDS data requires information about the social network size of each respondent (degree) in order to adjust for the participant’s differential probabilities of inclusion. The social network size of the study’s participants was assessed by asking them how many Somalis living in Oslo each of them knew who they could meet and talk to at least twice a month (strong link).

### Analysis

Weighted population estimates and a 95% confidence interval were calculated using a Respondent Driven Sampling Analysis Tool (RDSAT) v. 6.0.1, whereas SPSS was used for univariate and multivariate analyses using RDSAT exported weighted data.

Whether equilibrium was achieved on key variables, and the number of waves needed for each variable to reach equilibrium, was also calculated. Generally speaking, equilibrium is an indication that the final sample is independent of the non-randomly selected seeds [29], with the variables used in assessing equilibrium in this study being age, years of stay in Norway and support for the continuation/discontinuation of FC.

Descriptive analyses were conducted using a Chi-square test to estimate proportional differences, although where the cell sizes were small (expected cell counts of less than five), Fisher’s exact tests were used instead. Univariate logistic regression procedures were used to examine associations between the outcome of interest and each independent variable. A separate multivariable logistic regression model was built for each independent variable that was statistically significant (p < 0.05) in the univariate models, and adjusted for gender, age, years of residence in Norway and education. The degree of association was measured by a 95% confidence interval (CI) and odds ratio (OR).

## Results

Of the study participants, 50.5% were men and 49.5% were women (Table 1), and the age of the participants ranged from 18 to 70 years, with a mean age of 29 (SD 10.4). From an educational perspective, 12.3% of the study participants had a university education, 56.1% had a secondary education, 22.6% had a primary education and only 6.6% had no formal education. According to the participants’ length of residence in Norway, 50.4% of the total participants resided in Norway ≤9 years. However, this differs by gender. While 43% of the male respondents resided in Norway ≤4 years, nearly 50% of the females lived in Norway ≥10 years. The difference in years of residence in Norway between men and women was statistically significant (P < 0.001) (Table 2).

**Table 1:** Demographic characteristics of study participants

**Variables**	**N**	**(%)**
**Gender**		
Male	107	50.5
Female	105	49.5
**Total**	212	100
**Age**		
≤25	95	44.8
26.35	63	29.7
36-45	40	18.9
46+	11	5.2
Total	209	98.6
**Marital status**		
Single	78	36.8
Married	89	42
Divorced	34	16
widowed	6	2.8
Total	207	97.6
**Education**		
University	26	12.3
Secondary	119	56.1
Primary	48	22.6
No formal education	14	6.6
**Total**	207	97.6
**Years of residence in Norway**		
≥15	39	18.4
10-14	47	22.2
5-9	57	26.9
≤4	62	29.2
**Total**	205	96.7

**Table 2:** Gender differences in the knowledge about the health effects of FC and the attitude towards the practice

**Variables**	**Women (%)**	**Men (%)**	**P-value**
**Education**			
University	4.5	11.8	P < 0.02*
Secondary	46.4	60.2	
Primary	38.4	21.5	
**No formal education**	10.7	6.5	
Marital status			
Single	31.5	53.8	P < 0.03*
Married	40.5	32.3	
Divorced	26.1	9.7	
Widowed	1.8	4.2	
**Years in Norway**			
>14	20.2	14	P < 0.001*
10-14	29.4	19.4	
5-9	34.8	23.6	
0-4	15.6	43	
**Knowledge of health effects of FGM**			
Infection			
Yes	95.1	95.7	P < 0.594
No	4.9	4.3	
**Complications in child birth**			
Yes	92.5	95.8	P < 0.293
No	7.5	4.2	
**Urine retention**			
Yes	90.2	93.4	P < 0.316
No	9.8	6.6	
Yes	90.3	91.9	P < 0.456
No	9.7	8.1	
**HIV transmission**			
Yes	95.3	96.4	P < 0.496
No	4.7	3.6	
**Attitude towards FGM**			
None of FC forms are religious duty	70	47.3	P < 0.002*
One/both forms are religious duty	30	52.7	
**Does FC Preserve the dignity of girls?**			
None of the forms do	73.6	55.4	P < 0.006*
One/both forms do	26.4	44.6	
**Does FC prevent premarital sex**			
Yes	71.4	64.5	P < 0.183
No	28.6	35.5	
**Necessary for marriageability of girls**			
Yes	58.6	58.1	P < 0.528
No	41.4	41.9	

*Statistically significant at P < 0.05.

Regarding the equilibrium of major variables such as age, years of stay in Norway and support towards the continuation/discontinuation of FC, equilibrium was achieved at the 3^rd^ and the 3^rd^ and 5^th^ waves, respectively. As for homophily, the participants were randomly recruited for most of the major variables, i.e. at least 70% of the time, the recruitment was randomly mixed for the trait, “years of stay in Norway.”

Regarding the circumcision status of the female participants, 13% were left uncircumcised, 19.6% were circumcised using the *Sunna* form and 64.7% were circumcised with the Pharaonic form. Out of a total of 10 female participants who came to Norway at the age of seven or lower, nine (90%) were left uncircumcised, while only one (10%) was circumcised, though where this circumcision took place was not asked. Regarding people’s intention to circumcise their daughters, 81% had no intention to circumcise their daughters, whereas 18% did intend to do so, with the vast majority of those having lived in Norway ≤4 years, and more likely to be male.

Almost 60% of the male participants preferred to marry uncircumcised women, while 40% preferred circumcised women to be their wives. Those who chose circumcised women were more likely to have lived in Norway ≤9 years, with the majority (60%) living in Norway ≤4 years.

There were no significant differences in relation to knowledge of the health effects of FC between those who supported the continuation/discontinuation of FC, with both groups having an equal knowledge that FC leads to bleeding, infections, complications in child birth, difficulty in urination and the possible transmission of HIV.

Nonetheless, 70% of the people supported the discontinuation of all forms of FC, and a demographic analysis shows that there is no statistically significant association between support towards continuation of FC and age, level of education and years of stay in Norway. Even so, people who lived in Norway ≤4 years were twice as likely to support the continuation of FC compared to those who lived in Norway >14 years, though this difference did not reach a statistically significant level (OR = 2.37 CI: 0.94-6.01). In contrast, a significant association was observed between gender and support towards the continuation of FC, with women being less likely to support the continuation of FC (OR 0.17, CI: 0.091-0.34) (Table 3).

**Table 3:** Association between support towards discontinuation of FC and demographic variables

	**Weighted %**		**Crude**	
**Variables**	**Support discontinuation of all forms**	**Support continuation of one form or both forms**	**OR**	**95% CI**
**Gender**	(%)	(%)		
men	51.6	48.4	1.00	1.00
women	85.7	14.3	0.17	0.091-0.34
**Age**				
≤25	69.1	30.9	1.00	1.00
26-35	75.8	24.2	0.72	0.35-1.50
36-45	64.9	35.1	1.25	0.56-2.78
46+	63.6	36.4	1.12	0.30-4.21
**Education**				
University/College	56.3	43.8	1.00	1.00
Secondary	75.9	24.1	0.44	0.15-1.33
Primary	65.1	34.9	0.75	0.24-2.32
No education	61.1	38.9	0.93	0.24-3.71
**Years of residence in Norway**				
>14	75	25	1.00	1.00
10-14	86	14	0.48	0.16-1.48
5-9	65	35	1.61	0.63-4.11
≤4	56.9	43.1	2.37	0.94-6.01

By contrast, for those who perceived FC as a religious requirement (OR 47.3, CI: 17.6-126.7), the S*unna* form was a positive cultural factor (OR 14.03, CI: 6.72-29.29), protected against pre-marital sex (OR 2.64, CI: 1.41-4.96) and FC was a prerequisite for a trustworthy marriage (OR 2.02, CI: 1.10-3.71), had significantly higher odds of supporting the continuation of FC compared to their corresponding groups in a univariate analysis. However, most of these variables lost their significance when adjusted for age, gender, education and years of residence in Norway. That FC is a religious requirement remained significant, as those who believe that FC (particularly the S*unna* form) is a religious requirement had 41 times higher odds of supporting its continuation compared to those who believe that FC is not required by Islam (OR 40.9, CI:10.22-164.1) (Table 4).

**Table 4:** Univariate and multivariate logistic regression analysis for the association of support in the continuation/discontinuation of FC and the attitude of people towards the practice

	**Weighted %**		**Crude**	Adjusted*
**Attitude variables**	**Support Discontinuation of all forms**	**Support Continuation of one form or both forms**	**OR (95% CI)**	**OR (95% CI)**
**Is FC required by your religion?**				
FC is not a religious requirement	95.9	4.1	1.00	1.00
FC is a religious requirement	31.7	68.3	**47.3(17.6-126.7)**	**40.9(10.22-164.1**
**Is FC a positive cultural aspect?**				
FC is a harmful cultural aspect	89.6	10.4	1.00	1.00
Yes, the Sunna form is a positive cultural aspect	38.2	61.8	14.03(6.72-29.29)	3.40(0.99-11.67)
**Does FC lead to a trustworthy marriage?**				
No	76.5	23.5	1.00	1.00
Yes	61.2	38.8	**2.02(1.10-3.71)**	1.17(0.33-4.16)
**Does FC protect against adultery?**				
No	77	23	1.00	1.00
Yes	55.4	44.6	2.64(1.41-4.96)	2.48(0.61-10.0)
**Does FC preserve the dignity of the girls?**				
No	78.8	21.2	1.00	1.00
Yes	54.3	45.7	3.11(1.66-5.81)	1.78(0.40-7.95)

*Adjusted for age, gender, education and years of residence in Norway.

## Discussion

Twenty-two years since the major migration from Somalia to Norway began, this quantitative study investigates whether or not the attitudes toward FC among Somali immigrants have improved in favor of its abandonment. The findings reveal that 70% of the study population supported the discontinuation of all forms of FC, which is consistent with recent qualitative findings that showed that Somalis in Oslo are on the brink of abandoning all forms of FC [2]. A sustained decline in the prevalence of FC has been reported to be the ultimate indicator of a change in behavior towards FC [28], and the fact that 90% of the girls who came to Norway at the age of ≤7 were not circumcised is therefore an indication of an abandonment of FC among Somali immigrants living in Oslo. A prior study in London indicated that Somali girls who came to the UK before or at about the usual age of circumcision were more likely not to be circumcised than those who did not [6]. Additionally, the finding that 81% of the study participants had no intention to circumcise their daughters indicates that Somalis living in Oslo have a tendency towards abandoning the FC tradition, with previous studies in both the UK and Sweden also reporting a similar result [6,36]. While there has been a large amount of research that has produced sufficient evidence to support the plausible abandonment of FC among Somali immigrants [2,4,6,37], others argue that FC is a traditional norm deeply imbedded within the social culture of the Somali people, which could take generations to abandon [38]. However, by reflecting the introduction of FC in Sub-Saharan Africa, there is evidence that the spread of the practice has taken place very quickly [24]. If circumstances similar to this occur and help facilitate the rapid abandonment of FC in the context of migration, there are a number of reasons to believe that the practice can also be abandoned very quickly.

Firstly, infibulation was almost absent in the southern Sab clans in Somalia in the early 19^th^ century, while it was at almost 95% in the northern Noble clans. The southern clans later adopted infibulation by emulating the Noble clans in order to gain status [18]. In accordance with this, Mackie has mentioned that it took 20 years from the introduction of FC in Nyertete in Sudan until it reached a universal state [26]. The Nyertete communities in Sudan, as well as the Sab clans in Somalia, both adopted the practice of FC quickly in order to ensure that their girls qualified for marriage by Noble clans and to ensure the economic security of their daughters. This indicates that the main motivational factor for the practice is that parents want what is best for their children, and once an alternative condition emerges within a given community and parents recognize that their daughters will be worse off with circumcision, they will also abandon the practice [24]. A recent qualitative study in Oslo shows that being circumcised is no longer a status-related development among Somalis in Oslo due to the fact that uncircumcised young girls are much more likely to attract boyfriends, and have a higher chance to be married than their circumcised counterparts [2]. If FC provided status in Somalia and was adopted in order to ensure the economic security of girls in Norway, where FC is no longer status-related, but instead a crime and stigmatizing, the Somali immigrant’s tendency to abandon the practice would not be unexpected.

Secondly, the type and frequency of FC in Somalia are predicted by region of residence and not by the birthplace of the parents [18]. For example, people who moved from Northern Somalia, where infibulation was common, to Southern regions, where the milder forms of FC were predominant, adopted the practice common to their new residence. Hence, the abandonment of the practice by Somali immigrants who resided in the West, where FC is a crime, is not a surprising move. Furthermore, tribal facial scarring is a common practice in certain communities in Southern Sudan, where it represents beauty and status, but is a disgrace among educated and urbanized youths from practicing communities residing in the capital of Khartoum, where facial scarring is not practiced. In a similar manner, FC represents status in Somalia, but in regard to Somalis in Oslo, circumcised girls often conceal their circumcision status from boys because Westernized Somali boys often prefer uncircumcised girls for marriage or relationships over circumcised girls [2]. Consequently, migration to the West is highly likely to be a dead-end for FC among the Somali community.

According to the modified stages of change theory, people abandon the practice when they find a motivation to do so and have the ability to act upon their decision, i.e. when their social context supports, promotes or at least accepts abandonment [25]. This may apply to Somalis in Norway because the social pressure that perpetuated the practice in Somalia no longer exists in Norway, where being uncut is the norm, thus perpetuating a supportive environment for change. Furthermore, Shell-Duncan has argued that in certain situations people may sometimes abandon the practice even if they are personally in favor of its continuation, e.g. when other decision makers or social pressure force them to stop the practice, or where there is an enforced law that forbids the practice and there is a fear of prosecution for breaking this law [25]. This could also be true in Oslo since the practice of FC is a high-profile crime, and every person is informed about it as soon as he/she arrives in the country. Based on external pressure, there are uncertainties surrounding the sustainability of such a change. However, the positive side of such pressure is that when uncut generations who are born or brought up in Norway become parents, they will most likely automatically reject the practice since they were not cut, and have never experienced the social pressures that sustain the practice; therefore, they will have no reason to cut their daughters’ genitals.

Although FC is an old cultural tradition that every Somali girl undergoes in one form or another, culture is not static, but instead a dynamic process that changes with the circumstances that surround it. Factors such as migration, social context and the level of education can amend a culture, and play a significant role in reshaping a community’s norms, behaviors and values [39]. Once migration occurs, attitudes toward continuing female circumcision may change [40]. For example, when Somali immigrants arrive in Norway, they experience different perceptions of parental practices, women’s status and role in society, and through intense awareness campaigns they understand more on the health and religious aspects of the practice, which may lead to a re-evaluation of initial perceptions concerning female circumcision [2]. In accordance with this, a prior study reports that the longer that Somalis stay in exile, the higher the likelihood that they will abandon FC [6]. The argument is that where FC is not a social norm and is not associated with social status by the majority culture, some of the social pressures influencing its continuation will “slowly but surely” disappear. A recent qualitative study in Oslo reports that having undergone FC no longer gives status in Norway, but instead disgrace [2], which strongly challenges the initial perceptions concerning the importance of female circumcision such as status, religious duty or being part of a good culture.

In this study, women were less likely to support the continuation of the practice compared to men, with a similar trend being found in Somalia, as well as with Somali immigrants in London [6]. In addition, approximately 40% of men in Oslo preferred a circumcised woman as a wife. The reported gender difference in attitudes toward the practice could be related to the fact that the vast majority of male respondents in this study resided in Norway ≤4 years, while nearly 50% of female respondents resided in Norway ≥10 years. Thus, male respondents might not have benefitted fully from awareness campaigns compared to women. The other possible explanation is that FC is often considered a female issue in Somalia, though this tradition is not limited to only Somalia. Even in the West, the issue of FC is a female issue from the grassroots to the professional levels, and the organizations that deal with FC are primarily women’s organizations, with their grassroots audiences being other females. As a result, men are often deprived of information regarding the practice.

Fortunately, while many other immigrants from FC-practicing countries are very secretive and try to avoid talking about FC or expressing the reality of FC in their communities, many Somalis are outspoken and openly accept that FC was part of their culture, a harmful culture that they are committed to fighting against [41]. Nevertheless, a prior study reveals that some Somali immigrants have expressed anger that their efforts to deal with FC have never been acknowledged, and that they have been highly stigmatized for having FC as part of their culture, which is the very culture they are trying to fight against [41]. While the present study and others [2,6,36,37] clearly show that FC abandonment among Somali immigrants is a matter of reality, the successful community programs that may have contributed to this change are rarely acknowledged. In general, people are more likely to promote a new behavior when they are rewarded for doing so. Instead of rewarding and promoting the community efforts that led to such a successful change, if more “culturally aggressive” measures are imposed on communities, i.e. the “genital checkup of girls,” community motivations toward the change may be impeded. We therefore recommend that more efforts should be dedicated toward understanding, acknowledging and promoting the grassroots efforts that have proven to be successful in changing people’s attitudes toward the practice.

The important limitation of our study is its cross-sectional design, thereby making it difficult to establish the causes. Moreover, all of the variables were self-reported, with a distinct possibility of both under- and/or over reporting. For instance, self-reported types of FC may not be a reliable source of information; however, a validation study comparing self-reported types of FC among Somali women and the reality obtained through clinical examinations is under way. The practice is illegal in Norway, which may affect some people’s decision to report their position towards the continuation of this illegal practice. Nonetheless, this was minimized by employing research assistants who were trusted and not associated with any authority by the study participants.

## Conclusions

Although the study demonstrates promising results about a change in attitudes toward the abandonment of the practice among Somali immigrants in Oslo, intervention measures targeting the 30% of the people who are still sympathetic to the continuation of the practice are required. Accordingly, since religion was a significant factor behind the positive attitude towards the practice, religious intervention should be prioritized.

Men are more likely to have a positive attitude towards the practice than women; hence, awareness campaigns should equally target men, as it is widely known that without men’s involvement in eradication programs, the efforts towards the abolishment of the practice may have little chance of success. In addition, people with a positive attitude towards the practice are more likely to be people who have resided in Norway for a few years. Awareness programs in the early phase of immigrants’ arrival could help improve their attitude towards the practice in subsequent years.

Community efforts have a paramount importance in the ultimate eradication of FC. Therefore, communities should be empowered in such a way that helps them to take responsibility for their own matters, including the eradication of FC and other practices that are harmful to community health. Accordingly, people who have changed their behavior and left their girls uncut should be equipped with more knowledge and be morally supported so that they recruit other people, including their neighbors in Oslo, as well as their family members and networks in Somalia, to help abandon this practice.

## Competing interests

The authors declare that they have no conflict of interest.

## Authors’ contributions

AG wrote the research protocol, initiated the fieldwork, did the data analysis and drafted the manuscript. E J was involved in the data-analysis, editing and commenting on the manuscript. JS was involved in the write-up of the research protocol, the fieldwork, the data analysis and the drafting of the manuscript. All authors read and approved the final manuscript.

## Pre-publication history

The pre-publication history for this paper can be accessed here:

http://www.biomedcentral.com/1471-2458/12/697/prepub
